# Altered structural brain networks at term-equivalent age in preterm infants with grade 1 intraventricular hemorrhage

**DOI:** 10.1186/s13052-020-0796-6

**Published:** 2020-04-10

**Authors:** Jong Ho Cha, Yong-Ho Choi, Jong-Min Lee, Joo Young Lee, Hyun-Kyung Park, Jinsup Kim, Il-Kewon Kim, Hyun Ju Lee

**Affiliations:** 1 Department of Pediatrics, Hanyang University College of Medicine, Seoul, Hospital, 222-1 Wangsimni-ro Seongdong-gu, Seoul, 04763 South Korea; 2 Department of Biomedical Engineering, Hanyang University, Seoul, South Korea; 3 Division of Neonatology and Developmental Medicine, Seoul Hanyang University Hospital, Seoul, South Korea

**Keywords:** Diffusion tensor imaging, Preterm, Intraventricular hemorrhage (IVH), Brain network

## Abstract

**Background:**

Preterm infants are at risk for structural disruption of brain connectivity due to perinatal complications encountered during the fetal and neonatal periods. This study aimed to investigate the development of connectivity using diffusion tensor imaging at near-term age and the effect of grade 1 intraventricular hemorrhage on it.

**Methods:**

A total of 86 infants (55 preterm infants, 24 full-term infants) without apparent brain injury underwent diffusion magnetic resonance imaging (MRI) between 36 and 41 weeks post-menstrual age. The diffusion-MRI based connectomics were constructed from 64-segmented regions by using the Johns Hopkins University neonate atlas and were weighted with fractional anisotropy. The connectomes were quantified in the structural networks and investigated using network metrics, such as the clustering coefficient, local efficiency, characteristic path length, global efficiency, and small-worldness. We compared the differences in the brain networks of preterm infants with or without grade 1 intraventricular hemorrhage in binary and fractional anisotropy-weighted (wFA) connectomes.

**Results:**

The 55 preterm infants had a mean gestational age at birth of 29.3 ± 4.1 weeks and the 24 term-born infants, 38.1 ± 1.1 weeks. A total of 13 of the 55 preterm infants (23.6%) were diagnosed with grade 1 intraventricular hemorrhage. The development of connectivity of the brain network in preterm infants without intraventricular hemorrhage was comparable at near-term age to that in term infants. The preterm infants with germinal matrix hemorrhage exhibited higher clustering (0.093 ± 0.015 vs. 0.088 ± 0.007, *p* = 0.027) and local efficiency (0.151 ± 0.022 vs. 0.141 ± 0.010, *p* = 0.025), implying the potential for segregation. However, the preterm infants with intraventricular hemorrhage revealed a longer path length (0.291 ± 0.035 vs. 0.275 ± 0.019, *p* = 0.020) and lower global efficiency (3.998 ± 0.473 vs. 4.212 ± 0.281, *p* = 0.048), indicating a decreased integration in the wFA connectivity matrix than those without germinal matrix hemorrhage, after correcting for gestational age, sex, bronchopulmonary dysplasia, and age at scan.

**Conclusion:**

Grade 1 intraventricular hemorrhage in preterm infants may enhance the capacity for local information transfer and the relative reinforcement of the segregation of networks at the expense of global integration capacity.

## Background

Advances in neonatal intensive care have markedly enhanced the survival rate and decreased the rates of intraventricular hemorrhage (IVH) in preterm infants [1, 2]. Although previous efforts have helped in the declining incidence of intraventricular hemorrhage and its neurological sequelae, 40% of very low birth-weight infants still show language, cognitive, and behavioral impairments [3–5]. Research on the capacity of structural brain networks elucidated with diffusion magnetic resonance imaging (MRI) to predict functional impairments in very preterm infants has provided insight into the relationship between brain network topology and neurodevelopment. The diagnostic utility of brain imaging has prompted clinicians to explore the connectomes in infants and has considerably advanced the understanding of their neural circuits [6].

Preterm infants are at risk for structural disruption of brain connectivity due to the perinatal complications encountered during the fetal and neonatal period. Research has been conducted on the value of structural brain networks ascertained with diffusion MRI to predict the functional impairments and neurodevelopmental outcomes in very preterm children [7, 8]. Relative to full-term neonates, preterm neonates exhibit a decreased clustering coefficient, increased characteristic shortest path length, [6, 9] and decreased local and global efficiencies [10–12]. Our previous study on preterm infants has revealed higher small-worldness in preterm babies when compared to full-term controls at a term-equivalent age [13].

IVH is an important cause of neurological and cognitive complications and can significantly impair brain development [14]. Several recent studies suggest that even germinal matrix hemorrhage of a low-grade IVH can cause neurodevelopmental impairment in the future [15, 16]. However, the mechanism of the organization of the images of abnormally integrated and segregated brain networks in preterm infants with grade 1 IVH during early development remains poorly understood. Furthermore, whether the reorganization of structural brain networks is independently affected by grade 1 IVH remains unclear. This study used advanced MRI analysis, including diffusion tensor imaging (DTI)-based connectomes, to investigate grade 1 IVH-induced alterations to the preterm structural brain network at near-term age.

## Methods

### Study population

This study is part of a prospective observational cohort involving the postnatal follow-up of preterm infants at Hanyang Inclusive Clinic for Developmental Disorders in Hanyang University College of Medicine between December 2016 and June 2018. The institutional review board at Hanyang University Hospital approved the study protocol and scanning procedures, and informed consent was obtained from the parents of all children included in this study. The inclusion criteria for preterm infants consisted of the following: birth at < 35 weeks of gestation without major congenital malformations, no evidence of intraventricular or intracranial hemorrhage greater than grade I, no evidence of intrauterine growth retardation, and availability of MRI at near term age between 36 and 41 weeks postmenstrual age (PMA). Neonates with grade 1 IVH were included in our study. All cranial ultrasound scans and subsequent MRIs were evaluated for brain injury by a single pediatric radiologist. Early cranial ultrasound scans were performed within 3 days of birth and at 1 and 3 weeks later. Subsequent MRI scans conducted at near-term age were assessed for brain injury in preterm infants, including IVH, overt white matter injury, and cystic periventricular leukomalacia (PVL). We excluded 14 infants with evidence of intraventricular or intracranial hemorrhage greater than grade I, 1 infant with cerebellar hemorrhage, 2 infants with punctate white matter lesions, and 11 infants with poor image quality due to motion artifacts.

We performed MRI imaging in healthy, term infants (between 37 and 42 weeks gestation) with uneventful deliveries from the same hospital, after informing the parents in writing and obtaining their consent. The 24 healthy, full-term infants with normal MRI and neurologic examination results were included as controls. The following exclusion criteria for full-term infants were used: prolonged intensive care (> 7 days), neonatal asphyxia, congenital infection, and congenital heart disease. Controls and preterm infants were imaged during natural sleep without sedation. Prenatal and neonatal data were prospectively recorded, including maternal details, gestational age (GA), birth weight, delivery type, sex, head circumference at scan, germinal matrix hemorrhage, culture-proven sepsis, necrotizing enterocolitis, and bronchopulmonary dysplasia (BPD). IVH was determined using the grading system established by Papile et al. [17]. and was classified into unilateral or bilateral grade 1 IVH based on the new scoring system by Al-Mouqdad et al. [18]. The diagnosis and severity of BPD were defined by the need for supplemental oxygen at 28 days of age and at 36 weeks of GA [19].

### MRI imaging

T1- and T2-weighted imaging and DTI scans were acquired with a 3.0 T MRI scanner (Philips Real Time Compact Magnet 3.0-Tesla MRI system, Achieva 3.0-Tesla X-series) and a 16-channel SENSE head coil. The MRI data were obtained at the age of 36–41 weeks PMA in preterm infants and at 37–40 weeks PMA in full-term infants. The T1- and T2-weighted images were obtained with sagittal T1 Turbo field-echo sequences (repetition time [TR]/echo time [TE] 8.2/3.8 ms) and Turbo spin-echo (TR/TE 4800/90 ms) sequences, respectively. The DTI sequence parameters consisted of the following: SENSE factor, 2; b value, 800 s/mm^2^; number of diffusion gradient directions, 15; TR/TE, 5243/76 ms; 40–50 axial slices; 2.0-mm thickness; field-of-view, 150 mm; matrix size, 1.97 × 1.97; total acquisition time, 6.5 min.

### Diffusion processing

**Fig. 1:**
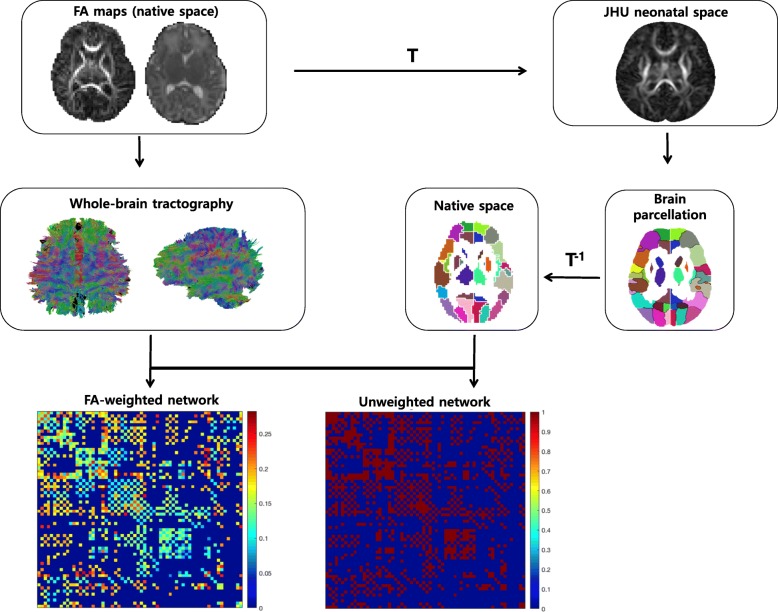
The flowchart of structural network construction. The fractional anisotropy (FA) image of each neonatal subject in the native space was registered to the FA template in the John Hopkins University (JHU) atlas space with the transform T. The JHU atlas labels (Brain parcellation) were inversely transferred to the native space with the inverse transform T^− 1^. With delineation of network edges and nodes in the native space, the structural networks were constructed

Diffusion-weighted imaging (DWI) data were preprocessed using the Diffusion Toolbox (FDT) from the FMRIB’s Software Library (http://www.fmrib.ox.ac.uk/fsl). The eddy current distortion and head motion for individuals were corrected using FMRIB’s Linear Registration Tool (FLRIT) [20, 21]. The Brain Extraction Tool (BET) provided by FSL was used to remove non-brain tissues [22]. The diffusion tensor in each voxel was then estimated using least-squares optimization [23]. The scalar map, including three eigen values (λ1, λ2, λ3), fractional anisotropy (FA), and mean diffusivity (MD) were subsequently obtained. Whole-brain deterministic diffusion fiber tracking was performed for each infant using quantitative anisotropy in DSI studio and was estimated using a streamlined algorithm in DSI studio (dsi-studio.labsolver.org) [24]. The FA threshold was set to 0.1, and the tracking turning angular threshold between the two streamlines was 45°. For the network construction, the definitions of the nodes and edges of the network were described as the two fundamental elements of a network necessary for the construction of an individual structural network. The network nodes were defined based on the Johns Hopkins University (JHU) neonate atlas [25]. We used estimated fiber tracts in the whole brain for edge definition and quantified connectivity between the cortical regions. If at least three fiber tracts were found between cortical regions, we considered the two regions to be structurally connected. The connectivity strength was then computed by using the average FA value along the fiber tracts [26, 27]. Finally, we constructed weighted and unweighted 64× 64 connectivity network matrices for each subject (Fig. 1). We introduced the following two different pipelines: binary and FA-weighted (wFA); the former shows whether the connection between regions exists, [28] while the latter presents the FA network normalized by the total FA weight of the whole-brain connectivity to independently assess the brain organization and to gauge the strength of the overall network [29].

### Network analysis

At a global level, connectomes were quantified in the structural networks and investigated using various network metrics, including integration measures, segregation measures, and small-worldness. Topological organizations of neonatal structural networks were characterized using the Brain Connectivity Toolbox in MATLAB (the Mathworks, Inc., Natick, Massachusetts, USA) [28]. The network integration refers to the mechanism behind the integration of discrete specialized information by the nodes in an efficient and rapid manner. The most popular network integration measures are characteristic path length (L_p_) and global efficiency (E_glob_). The shorter path length and increased global efficiency in structural networks suggest the potential integration for effective connections. In contrast to network integration, network segregation refers to the degree to which the network is organized as clusters of sub-networks. Segregation indicates the relative strength of within-network connections compared to integration, and the most popular measures of segregation are clustering coefficient and local efficiency. Finally, small-worldness reflects both integrated and segregated information processing. The descriptions and definitions of these network metrics for a given graph G with N nodes are provided in Table 1. All network metrics were calculated using the Brain Connectivity Toolbox (http://www.brain-connectivity-toolbox.net).

**Table 1:** Definitions and descriptions of network metrics

Network metrics	Definitions	Descriptions
Measures of segregation		
Clustering coefficient (Cp)	${L}_p=\frac{1}{N\left(N-1\right)}\sum \limits_{i\in G}\sum \limits_{j\in G,j\ne i}{L}_{ij}$	*L*_*ij*_ is the shortest path length between nodes *i* and *j*, *N* is the set of all nodes in the network *G*.
Local efficiency (Eloc)	${E}_{local}=\frac{1}{N}\sum \limits_{i\in G}{E}_{local,i}$	*E*_*local*, *i*_ is the local efficiency of node *i*.
Measures of integration		
Characteristic path length (Lp)	${C}_p=\frac{1}{N}\sum \limits_{i\in G}\frac{2\ast {E}_i}{k_i\left({k}_i-1\right)}$	*k*_*i*_ is the degree of node *i*, *E*_*i*_ is the number of edges that consists of the neighbors of node *i*.
Global efficiency (Eglob)	$$ {E}_{glob}=\frac{1}{N\left(N-1\right)}\sum \limits_{i\in G}\sum \limits_{j\in G,j\ne i}\raisebox{1ex}{$1$}\!\left/ \!\raisebox{-1ex}{${L}_{ij}$}\right. $$	It is inversely related to *L*_*p*_.
Other concepts		
Small-worldness (σ)	$\upsigma =\frac{L_p/{L}_{ran}}{C_p/{C}_{ran}}$	σ measures small-worldness. Cran and Lran were averaged from 100 matched random networks.

*Cran, clustering coefficient for a random network; Lran, characteristic for a random network*

### Statistical analyses

SPSS 21.0 (SPSS, Chicago, IL) software was used for statistical calculations. We used the Mann-Whitney U-test and Fisher’s exact test to compare the clinical variables between groups. The preterm infants were sub-categorized into three groups as extremely preterm (< 28 weeks), very preterm (28 to 32 weeks), and moderate to late preterm (33 to 36 weeks). Mean network parameters in the preterm sub-groups were compared using the analysis of variance and general linear models with sex, BPD, and age at scan as covariates. Analysis of covariance was performed to confirm the differences in networks between preterm and full-term infants. Additional statistical analysis between preterm infants with and without grade 1 IVH was performed using general linear models with GA, sex, BPD, and age at scan as covariates to compare the structural network metrics, including clustering, local efficiency, path length, global efficiency, and small-worldness.

## Results

The total population consisted of 55 preterm infants (26 males) with a mean GA at birth of 29.3 ± 4.1 (range 23–35) weeks and a mean age at scan of 37.2 ± 1.4 (range 36–41) weeks, and 24 term-born infants (18 males) with a mean GA at birth of 38.1 ± 1.0 (range 37–40) weeks and a mean age at scan of 39.8 ± 0.9 (range 37–41) weeks. Table 2 presents the demographic and clinical characteristics of both groups in the study. Thirteen of the 55 preterm infants (23.6%) were diagnosed with grade 1 IVH. Notably, 4 infants developed both grade 1 IVH, while 9 infants developed unilateral grade 1 IVH (Right 6 and Left 3). Compared to the full-term group, the preterm infants without grade 1 IVH underwent MRI significantly earlier (37.8 ± 1.7 vs 39.8 ± 0.9, *p* < 0.001) and featured a lower proportion of males (45.2% vs 75.1%, *p* = 0.028).

**Table 2:** Baseline demographic and clinical characteristics of the preterm and term infants included in the study

Variables	Preterm with GMH (*n* = 13)	Preterm without GMH (*n* = 42)	Term group (*n* = 24)	*P* value preterm without GMH vs. term	*P* value (with vs. without GMH)
GA, wk	27.4 ± 3.1	30.1 ± 3.8	38.1 ± 1.0	< 0.001	0.002
BW, g	909.2 ± 373.1	1373.5 ± 471.6	3142.9 ± 306.9	< 0.001	0.002
HC, cm	25.1 ± 3.5	27.4 ± 3.1	34.0 ± 1.1	< 0.001	0.004
Cesarean section	8 (66.7)	36 (85.7)	10 (41.7)	0.011	0.203
Male sex	7 (53.8)	19 (45.2)	18 (75.1)	0.028	0.068
HC MRI, cm	33.1 ± 1.2	33.9 ± 1.5	34.7 ± 2.3	0.079	0.068
Age at MRI scan, wk	37.2 ± 1.4	37.8 ± 1.7	39.8 ± 0.9	< 0.001	0.116
Culture-proven sepsis	3 (23.1)	6 (14.3)	–	–	0.428
NEC (requiring surgery)	1 (7.1)	1 (2.2)	–	–	0.925
TPN days	44.42 ± 28.16	30.61 ± 29.6			0.156
BPD	10 (76.9)	21 (50.0)	–	–	0.116

Data are the means ± SD or number (%)

*GA* gestational age; *BW*, birth weight; *HC* head circumference; *GMH* germinal matrix hemorrhage; *MRI* magnetic resonance imaging; *NEC* necroenteritis colitis; *TPN* total parenteral nutrition; *BPD* bronchopulmonary dysplasia

Relative to the preterm infants without grade 1 IVH, preterm infants with grade 1 IVH exhibited a low mean GA (27.4 ± 3.1 vs 30.1 ± 3.8, *p* = 0.002) and birth weight (909.2 ± 373.1 vs 1373.5 ± 471.6, *p* = 0.002). However, the age at the MRI scan and head circumference at age of MRI scan were not significantly different between preterm infants with and without grade 1 IVH. During the neonatal period, preterm infants with grade 1 IVH differed in that they exhibited a high rate of BPD (76.9% vs 50%, *p* = 0.116) and a longer duration of total parenteral nutrition days (44.42 ± 28.16 vs 30.61 ± 29.6, *p* = 0.156). This is associated with chronic intubation and poor nutritional support for proper brain growth; however, these differences were insignificant.

Table 3 shows the comparison of brain network parameters, including binary and weighted FA connectivity matrices in the following preterm sub-groups: extremely preterm (< 28 weeks GA, *n* = 21), very preterm (28–32 weeks GA, *n* = 18), and moderate to late preterm (33–36 weeks GA, *n* = 16). These exhibited no significant differences among preterm sub-groups in the statistical analyses, controlling for sex, BPD, and age at MRI scan as covariates in preterm infants. Table 4 shows the results of the comparison of brain network parameters, including binary and weighted FA connectivity matrices, among preterm infants with and without grade 1 IVH and full-term infants. Compared to the full-term group, preterm infants without grade 1 IVH underwent MRI significantly earlier (37.8 ± 1.7 vs 39.8 ± 0.9, *p* < 0.001) and featured a lower proportion of male infants (45.2% vs 75.1%, *p* = 0.028). The preterm neonates without grade 1 IVH exhibited slightly lower wFA clustering coefficients (0.088 ± 0.007 vs. 0.094 ± 0.007, *p* = 0.375), and wFA local efficiency (0.141 ± 0.010 vs 0.152 ± 0.014, *p* = 0.486) than full-term infants. However, the clustering, local efficiency, path length, global efficiency, and small-worldness in preterm infants without grade 1 IVH were comparable to those in full-term infants after adjusting for GA, sex, BPD, and age at scan. Remarkably, the preterm infants with grade 1 IVH exhibited higher clustering (0.093 ± 0.015 vs. 0.088 ± 0.007, *p* = 0.027) and local efficiency (0.151 ± 0.022 vs. 0.141 ± 0.010, *p* = 0.025), implying the potential for segregation. However, they exhibited longer path length (0.291 ± 0.035 vs. 0.275 ± 0.019, *p* = 0.020) and lower global efficiency (3.998 ± 0.473 vs. 4.212 ± 0.281, *p* = 0.048), indicating a decreased integration in the wFA connectivity matrix among preterm infants with grade 1 IVH than those without grade 1 IVH, after correcting for GA, sex, BPD, and age at scan. In contrast, we observed no significant differences in the binary network parameters with and without grade 1 IVH.

**Table 3:** Mean network value in preterm infants

Variable	Extremely preterm < 28 wks (*n* = 21)	Very preterm 28 to 32 wks (*n* = 18)	Moderate to late preterm 33 to 36 wks (*n* = 16)	*P* value among groups	Adjusted P^a^ among groups
Bi Clustering coef.	0.573 ± 0.024	0.556 ± 0.032	0.564 ± 0.024	0.201	0.471
Bi Local eff.	0.770 ± 0.021	0.759 ± 0.027	0.761 ± 0.021	0.305	0.701
Bi cPL	1.910 ± 0.090	1.882 ± 0.091	1.897 ± 0.046	0.583	0.684
Bi Global eff.	0.599 ± 0.023	0.606 ± 0.025	0.601 ± 0.012	0.573	0.501
Bi Small-worldness	1.543 ± 0.147	1.486 ± 0.177	1.462 ± 0.095	0.221	0.882
wFA Clustering coef.	0.091 ± 0.009	0.089 ± 0.013	0.088 ± 0.006	0.629	0.471
wFA Local eff.	0.145 ± 0.012	0.144 ± 0.020	0.140 ± 0.009	0.524	0.701
wFA cPL	0.283 ± 0.020	0.277 ± 0.034	0.275 ± 0.018	0.654	0.684
wFA Global eff.	4.085 ± 0.310	4.176 ± 0.454	4.226 ± 0.237	0.454	0.501
wFA small-worldness	1.464 ± 0.168	1.396 ± 0.187	1.361 ± 0.090	0.128	0.921

^a^controlling for sex, BPD, and age at MRI scan as covariates in preterm infants

Data are the means ± SD or number (%)

*Bi* Binary; *wFA* FA weighted; *SE* standard error; *cPL* Characteristic path length; *GMH* germinal matrix hemorrhage; *BPD* bronchopulmonary dysplasia

**Table 4:** Mean network value in preterm infants with IVH, preterm infants without IVH, and full term infants

Variable	Preterm with GMH (*n* = 13)	Preterm without GMH (*n* = 42)	Full-term (*n* = 24)	Adjusted P^a^ (preterm without GMH vs. term)	Adjusted P ^a^ (with vs. without GMH)
Bi Clustering coef.	0.572 ± 0.023	0.562 ± 0.028	0.557 ± 0.037	0.921	0.375
Bi Local eff.	0.769 ± 0.021	0.762 ± 0.024	0.755 ± 0.028	0.630	0.486
Bi cPL	1.910 ± 0.101	1.893 ± 0.708	1.870 ± 0.064	0.414	0.507
Bi Global eff.	0.599 ± 0.026	0.603 ± 0.019	0.609 ± 0.018	0.423	0.474
Bi Small-worldness	1.531 ± 0.166	1.488 ± 0.139	1.456 ± 0.104	0.718	0.748
wFA Clustering coef.	0.093 ± 0.015	0.088 ± 0.007	0.094 ± 0.007	0.375	0.027
wFA Local eff.	0.151 ± 0.022	0.141 ± 0.010	0.152 ± 0.014	0.486	0.025
wFA cPL	0.291 ± 0.035	0.275 ± 0.019	0.296 ± 0.038	0.507	0.020
wFA Global eff.	3.998 ± 0.473	4.212 ± 0.281	3.926 ± 0.420	0.474	0.048
wFA small-worldness	1.464 ± 0.179	1.391 ± 0.148	1.353 ± 0.099	0.748	0.479

^a^controlling for gestational age, sex, BPD, and age at MRI scan as covariates in preterm infants

Data are the means ± SD or number (%)

*Bi* Binary; *wFA* FA weighted; *SE* standard error; *cPL* Characteristic path length; *GMH* germinal matrix hemorrhage; *BPD* bronchopulmonary dysplasia

## Discussion

Developmental connectomics obtained from advanced neuroimaging techniques have emerged as an important tool for the characterization of the structural and functional brain connectivity during the early period of life in neonates. We explored potential alterations in structural network properties related to grade 1 IVH in preterm infants. The measures of structural segregation, such as modularity and local efficiency, were observed to increase, while global efficiency decreased in preterm infants with grade 1 IVH compared with those without grade 1 IVH, indicating a specialized reinforcement of network segregation at near-term age.

Previous studies have reported the effects of grade 1 IVH on the developmental white matter microstructure in preterm infants. Morita et al. [30] observed an association between grade 1 IVH in preterm infants and impaired cerebellar white matter from DTI images. Additionally, a study reported that the FA values in the corpus callosum, limbic pathway, and cerebellar tract are decreased in preterm infants with grade 1 IVH [31]. These infants with grade 1 IVH also had a poor development score at 24 months of corrected age. However, Payne et al. [32] exhibited that low grade intraventricular hemorrhage was not associated with adverse neurodevelopmental outcomes at 18 to 22 months in 1472 infants who were born before 27 weeks of GA. A previous study observed that except for grade 4 IVH, low grade IVH does not make a significant impact on the developmental outcome at 8 years of age in 270 infants who were born under 28 weeks of GA [33]. Despite many previous beliefs that it might not have any significant consequence, our study revealed that grade 1 IVH can alter the overall brain connectivity in grade 1 IVH.

The beginning of the development of the microstructure of white matter connections and the emergence and establishment of small worldness reportedly occurs throughout 30 PMA [6, 9, 10, 26]. When compared with preterm infants, the neural structural networks in full-term infants generally becomes more integrated with decreases in path length and increases in the global efficiency during the first phases of connectome development. Song et al. [34] suggested that increases in the global and local efficiencies indicate the development of both long distance and local connections in a weighted network matrix, which enable the brain to exchange information efficiently. This would further suggest that both integration and segregation processes increase with the age at which the scan is obtained, reflecting subtle changes in the neonatal brain. After early phases of the neonatal period, decreases in the clustering coefficient, modularity, and, hence, decreases in local efficiency as measured with diffusion MRI have been observed from the age of 6 months to 2 years in full-term infants [12, 35]. Evaluating the growth processes concomitant with the brain network shift from segregation to integration through the first 2 years of life, a few studies have reported that networks become more efficient in transferring information, indicating the improved integration in the full-term brain [11, 35]. We focused increasingly on normative preterm infants who were discharged successfully with no evidence of greater than grade 1 IVH. These results might reflect compensation-related network changes in preterm infants with grade 1 IVH, suggesting altered global efficiency and the relative preservation of segregated local efficiency in the structural brain networks during the rapid developmental period. In this study, the incidence (23.6%) of grade 1 IVH was relatively high in preterm infants less than 35 weeks, compared to that of a previous study by Bolisetty et al., in which 21.3% of preterm infants less than 28 weeks of gestational age were diagnosed with low grade IVH. These findings reflect that the preterm infants with grade 1 IVH in this study exhibited a low GA (27.4 ± 3.1 weeks) and birth weight (909.2 ± 373.1 g), involving higher incidence of IVH. Furthermore, we assume that the incidence of low grade IVH in this study was overestimated by exclusion of the infants with additional white matter injuries, cerebellar hemorrhage, and high grade IVH.

We explored the significance of the weighted networks and the differences between binary and FA-weighted networks. Discrepancies between unweighted or weighted connectome pipelines might be attributed to the differences in the speed at which networks change during dynamic developmental phases along with the abundant formation of new connections. To a lesser extent, differences in edge weights may account for the discrepancies. Compared to the binary approach, the FA-weighted approach could be more sensitive to the network organization of atypical early neural development in preterm infants than to the early development of the full-term brain [36]. Our observations by means of the FA-weighted matrices obtained from the diffusion MRI-based brain network indicated that the measures of structural segregation, such as clustering and local efficiency, augmented in preterm infants with grade 1 IVH; however, binary measures remained insignificant.

Ball et al. [27] demonstrated that, relative to term-born infants, preterm infants exhibit disrupted network capacities in both cortical-subcortical and cortico-cortical connections, possibly effected by the environmental stress induced by premature extra-uterine life on early network development during the neonatal period. After birth, the positive environmental motivation improves the integration capacity via the improvement of long-range connections. However, most preterm infants in the neonatal intensive care unit undergo an inevitable stressful environment or prematurity-related morbidity. Studies have shown the value of an optimal balance between integration and segregation in developmental connectome models after preterm birth with perinatal morbidity [8, 10]. With development, the balance between the integration and segregation of networks tends to be optimized with remarkable reorganization, resulting in maximized resilience against prematurity-associated pathology. Our previous study identified that the BPD is a critical factor in brain growth, which influences impaired white matter and cerebellar development in preterm infants at term-equivalent age [37]. We excluded the infants with intrauterine growth retardation and corrected for GA and BPD, which can negatively impact the neonatal brain growth and neurodevelopmental outcome, although not statistically different in the two groups. Despite the importance of the influence of prematurity-related morbidity on neurodevelopment, its correlation with grade 1 IVH has not been widely discussed. The prediction of the effect of grade 1 IVH on neurodevelopmental outcomes among preterm infants is currently among the clinical challenges of standard MRI studies, which are hampered by the limited capacity to evaluate neural reorganization processes and estimate differences in the architecture of structural brain networks. In the present study, after correcting for GA, sex, and BPD, the early development of grade 1 IVH led to a decrease of global efficiency and increase of local efficiency in structural brain networks compared to those of infants without grade 1 IVH. We identified aspects of network organization that mediates the effect of grade 1 IVH, which have revealed that such an organization reflects the increase of altered clustering at the expense of a global efficiency. Our findings provide insight to improve the understanding of how grade 1 IVH can subsequently alter later neurodevelopment using DTI-based connectomes. Accordingly, we aim to provide Bayley III scale data in the neurodevelopmental follow-up study to differentiate the preterm infants with abnormal brain development from relatively healthy preterm infants with normal brain development. This analysis may add novel findings to our results of grade 1 IVH-induced alterations to preterm brain network, implicating the involvement of cognitive and language development. The study is subject to a few limitations. First, our study contains a relatively small sample size when compared with studies performed with older age groups as the acquisition and analysis of neonatal MRI data are specialized and difficult tasks. Second, our diagnosis of grade 1 IVH was somewhat limited with the poor reliability, as all the cranial ultrasound scans were evaluated by a single pediatric radiologist. Furthermore, we could not include the repetitive longitudinal neuroimaging of the same cohort of preterm neonates and the implications for cognitive or language neurodevelopment.

## Conclusions

Despite these limitations, knowledge of the neonatal human connectome may provide insight into preterm development and the influence of grade 1 IVH on brain development at near-term age. Further follow-up studies based on structural brain networks should examine whether long-term neurodevelopment in preterm infants with grade 1 IVH is mitigated by delayed or altered integration and segregation compared to that in full-term infants.

## Data Availability

Reproducible materials described in the manuscript, including databases and all relevant raw data, are freely available to any scientist wishing to use them.
